# International Atherosclerosis Society Roadmap for Familial Hypercholesterolaemia

**DOI:** 10.5334/gh.1291

**Published:** 2024-01-25

**Authors:** Gerald F. Watts, Laney K. Jones, Mitchell N. Sarkies, Jing Pang, Samuel S. Gidding, Peter Libby, Raul D. Santos

**Affiliations:** 1 School of Medicine, University of Western Australia, Perth, Western Australia, Australia; 2 Departments of Cardiology and Internal Medicine, Royal Perth Hospital, Perth, Western Australia, Australia; 3 Department of Genomic Health, Geisinger, Danville PA, USA; 4 School of Health Sciences, Faculty of Medicine and Health, University of Sydney, Sydney, New South Wales, Australia; 5 Division of Cardiovascular Medicine, Department of Medicine, Brigham and Women’s Hospital, Harvard Medical School, Boston MA, USA; 6 Lipid Clinic, Heart Institute (InCor), University of São Paulo, São Paulo, Brazil and Hospital Israelita Albert Einstein, São Paulo, Brazil

**Keywords:** familial hypercholesterolaemia, roadmap, international, guidance, implementation practice, implementation strategies

## Abstract

Familial hypercholesterolaemia (FH), a common monogenic disorder, is a preventable cause of premature coronary artery disease and death. Up to 35 million people worldwide have FH, but most remain undetected and undertreated. Several clinical guidelines have addressed the gaps in care of FH, but little focus has been given to implementation science and practice. The International Atherosclerosis Society (IAS) has developed an evidence-informed guidance for the detection and management of patients with FH, supplemented with implementation strategies to optimize contextual models of care. The guidance is partitioned into detection, management and implementation sections. Detection deals with screening, diagnosis, genetic testing and counselling. Management includes risk stratification, treatment of adults and children with heterozygous and homozygous FH, management of FH during pregnancy, and use of lipoprotein apheresis. Specific and general implementation strategies, guided by processes specified by the Expert Recommendations for Implementing Change taxonomy, are provided. Core generic implementation strategies are given for improving care. Nation-specific cholesterol awareness campaigns should be utilized to promote better detection of FH. Integrated models of care should be underpinned by health policy and adapted to meet local, regional and national needs. Clinical centres of excellence are important for taking referrals from the community. General practitioners should work seamlessly with multidisciplinary teams. All health-care providers must receive training in essential skills for caring for patients and families with FH. Management should be supported by shared decision-making and service improvement driven by patient-reported outcomes. Improvements in services require sharing of existing resources that can support care. Advocacy should be utilized to ensure sustainable funding. Digital health technologies and clinical quality registries have special value. Finally, academic-service partnerships need to be developed to identify gaps in care and set priorities for research. This new IAS guidance on FH complements the recent World Heart Federation Cholesterol Roadmap.

Familial hypercholesterolaemia (FH), a co-dominant and highly penetrant monogenic condition that substantially elevates plasma LDL-cholesterol concentration, is a preventable cause of premature atherosclerotic cardiovascular disease (ASCVD), principally coronary disease [1]. FH affects up to 35 million people worldwide and has major public health implications, noting that only 10% of affected people are currently diagnosed and over 80% of those treated do not reach therapeutic LDL-cholesterol goals [2,3].

Several clinical guidelines and calls to action have addressed the gaps in care of FH, but little attention has been devoted to implementation science and practice [4,5]. The International Atherosclerosis Society (IAS) has developed an evidence-informed guidance that provides a systematic compendium of clinical recommendations for the detection and management of patients with FH, supplemented with implementation strategies to optimize the deployment of models of care [5].

The guidance is divided into detection, management and implementation sections. Detection covers screening, diagnosis, genetic testing and counselling. Management covers ASCVD risk stratification, treatment of adults and children with heterozygous and homozygous FH, management of FH during pregnancy, and use of lipoprotein apheresis. Specific and general implementation strategies, guided by the Expert Recommendations for Implementing Change [6]. are provided based on expert consensus (Figure 1). We summarize and contextualize the core strategies for implementing the clinical recommendations made in the recent IAS guidance, augmenting other guidelines.

**Figure 1:**
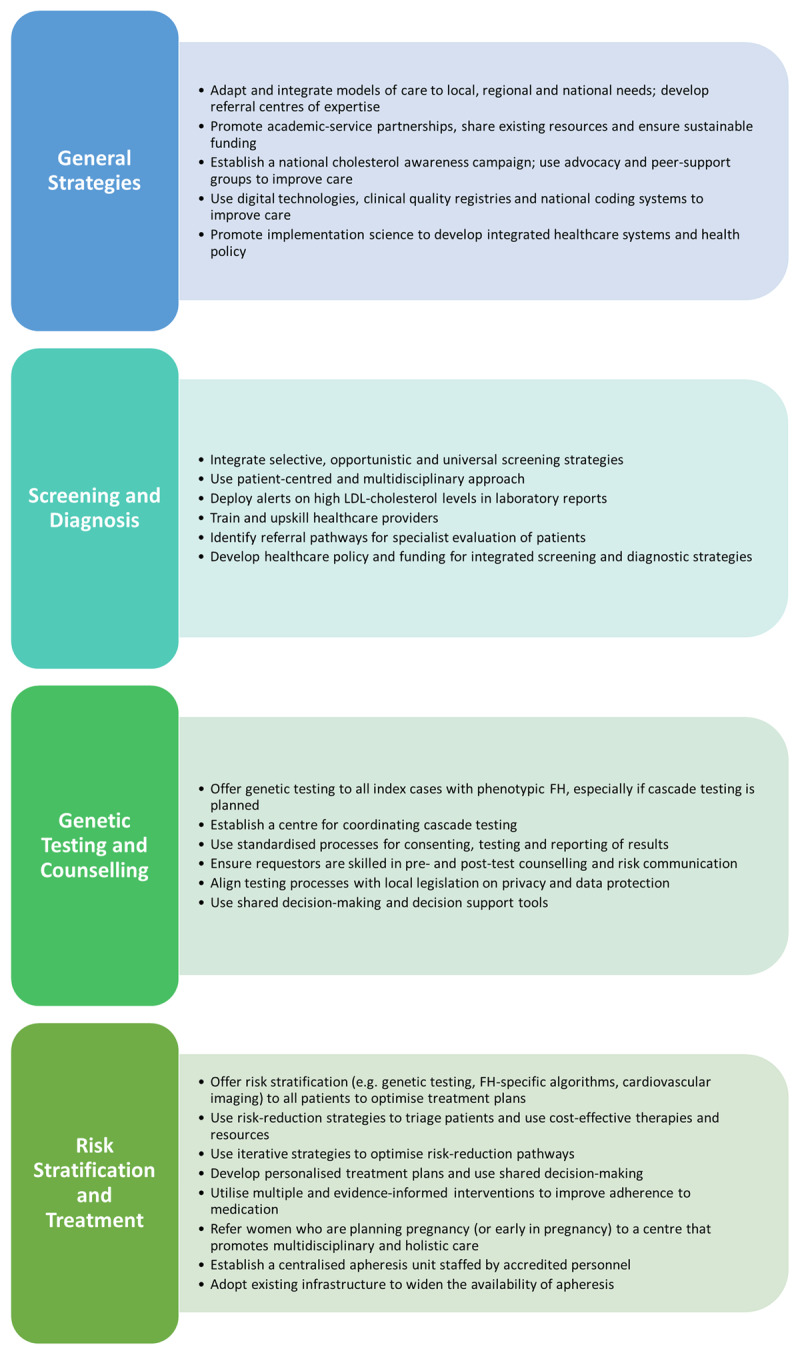
Overview of selected implementation strategies in the International Atherosclerosis Society guidance for best practice in the care of familial hypercholesterolaemia [5]. Abbreviations: FH: familial hypercholesterolaemia.

FH meets all criteria for the implementation of screening programs [1,2,4]. Implementation of such programs must also meet several criteria, such as feasibility, fidelity, adoption, access and reach, cost-effectiveness and sustainability. Screening strategies for FH (e.g. selective, opportunistic, universal), most practicably based on age-, sex-, and country-specific serum LDL-cholesterol above the 95^th^ percentile for the population, should be seamlessly integrated with each other, and supported by health policy and adequate funding [5]. A patient-centred and multidisciplinary approach, with a central role for general practice, should be followed. Digital technologies should be widely utilized and funded to search clinical databases and electronic health records for people at risk of FH. Clinical biochemistry services can play a key opportunistic role by utilizing alerts and comments on high LDL-cholesterol levels in laboratory reports. All health-care providers involved in detecting FH need training and skills in screening methods. Detection of FH needs to be coupled with appropriate cost-effective referral pathways for specialist assessment, the offering of genetic testing and optimal risk-reduction interventions.

The most accurate approach to diagnose FH is using genetic testing for pathogenic variants that impair LDL-receptor activity causing hypercholesterolaemia [1,4]. Genetic testing is not widely available and is underutilized, so that the diagnosis often relies on phenotypic criteria alone, the most widely employed being the Dutch and Simon Broome definitions [1,4], which need country-specific modifications. In children, the diagnosis of FH is made in the presence of elevated LDL-cholesterol and a positive family history of hypercholesterolaemia and premature coronary artery disease [7]; implementing screening of children for FH is a priority [8]. Because of a more florid clinical phenotype, the diagnosis of homozygous FH is clearer than heterozygous FH. All index patients with a phenotypic diagnosis of FH should ideally be offered genetic testing, especially if cascade testing is planned. Healthcare professionals involved in making a diagnosis of FH should know the local guidance on laws of genetic privacy. The diagnosis of FH in children and adolescents should ideally be made by a paediatrician with expertise in lipidology and in following local regulations on child protection. All patients diagnosed with homozygous FH should be referred to a specialist centre for further assessment and planning of care.

Cascade testing is the systematic testing of close relatives of a proband or index case with a genetic condition and is strongly advised for identifying FH. Cascade testing can be highly cost-effective and should ideally be centrally co-ordinated, and standardized processes followed for consenting, genetic testing and reporting of results. Requestors need to be skilled in counselling, genomics and FH. Digital tools and other practical resources should be employed to facilitate counselling and risk communication, and testing processes must be aligned with local legislation on privacy and data protection. Shared decision-making and decision support tools should be employed to enable genetic testing, the outcome of which should be included in national FH registries.

The risk of ASCVD in FH is driven mainly by the exposure to LDL-cholesterol, but also depends on behavioural, clinical and genetic factors [9]. Risk stratification methods, including cardiovascular imaging and FH-specific ASCVD prediction equations, can guide cost-effective therapeutic strategies. Risk stratification, supported by appropriate communication protocols, should be used to develop personalized treatment plans and to triage patients for referral to other services involved in the multidisciplinary care of FH. Digital health technologies and decision support systems should be used to facilitate risk assessment strategies. Telehealth services are critical for supporting risk assessment of patients in rural and remote regions. Risk stratification data need to be included in FH registries.

The treatment of FH using potent and available LDL-cholesterol lowering therapies alone and in combination (i.e. statins, ezetimibe, bempedoic acid, colesevelam, PCSK9 inhibitors) and management of other ASCVD risk factors is well supported by evidence [4,5] Lower LDL-cholesterol goals apply to those at higher risk; if treatment is started in childhood, LDL-cholesterol goals need not be as stringent as when started later in adulthood [4,5]. Treatment of homozygous FH is more difficult than heterozygous FH and involves newer therapies (inhibitors of microsomal triglyceride transfer protein and angiopoietin-like protein 3), lipoprotein apheresis, and very rarely liver transplantation [10]. The design of risk-reduction pathways needs to utilize cost-effective therapies and resources. Establishing a network of clinical centres to share experience and the education and upskilling of all health-care providers are essential for maintaining quality care. Iterative strategies with use of key performance indicators serve to optimize the effectiveness of care. Specific use of multidisciplinary care pathways, transitional services for adolescents, culturally appropriate approaches, and dedicated services for family planning and women during pregnancy are strongly recommended. Personalized treatment plans should be routinely employed using shared decision making and culturally sensitive communication. A special objective should be to identify patients not receiving guideline-directed therapy and facilitate treatment using multifaceted strategies, including advocacy and peer support. Non-adherence to management needs to be identified as a high-priority and addressed using multiple, evidence-informed interventions.

Lipoprotein apheresis is safe and highly effective adjunctive therapy for patients with homozygous FH and severe heterozygous FH [11]. However, the technique is inadequately utilized because of cost and logistic barriers. New injectable agents have decreased the need for apheresis in certain patients. Lipoprotein apheresis should meet local and regional needs of care, and be carried out in a centralized unit staffed by accredited personnel. Existing infrastructure should be adapted to increase the availability of apheresis. Multidisciplinary meetings should extend to all specialties that contribute to services. Apheresis-specific key performance indicators and patient-reported outcome and experience measures should be utilized to improve service delivery. Apheresis networks within and across countries are important to share educational, clinical and research experience. All patients should be consented for inclusion in a dedicated apheresis registry.

Core generic strategies should be adopted for implementing improved care of FH [5]. Nation-specific cholesterol awareness campaigns should promote better detection of FH [12]. Models of care should be integrated, underpinned by health policy and adapted to meet local, regional and national needs. The development of clinical centres of excellence is important for taking referrals across the continuum of care of FH. General practitioners should work seamlessly with multidisciplinary teams. All health-care providers must receive training and accreditation in essential skills for caring for patients and families with FH [5].

Management should be supported by shared decision-making and service improvement driven by patient-reported outcome and experience measures. Improvements in service models can accrue from sharing of existing resources across essential disciplines (e.g. cardiology, paediatrics, nursing, dietetics, pharmacy, transfusion medicine). Advocacy and peer-support groups should be utilized to ensure sustainable funding of services. Digital health technologies and clinical quality registries have special value in enhancing management. Finally, academic-service partnerships need to be closely developed to identify gaps in care and set priorities for clinical audits and research.

The expanding clinical knowledge on FH has set a precedent for countries to aspire to developing high-quality, integrated healthcare systems [4,5]. Implementation science provides the optimal approach to address the challenges required by the demands of changes in care made in the IAS guidance [5]. Economic, political and socio-cultural variations among countries may challenge universal adoption of these recommendations. Implementation strategies are context-dependent and need evaluation using implementation practice, at the heart of which is assessment of patient outcomes, a touchstone of effective healthcare policy.

The new IAS guidance provides a comprehensive roadmap for best clinical care of FH and will be useful for healthcare systems planning to initiate and develop services [5]. It synergizes with and augments the healthcare policy recommendations made in the recent World Heart Federation Cholesterol Roadmap [12]. These documents have the potential to provide optimal care for the greatest number of people with FH worldwide, but will need revision as new evidence rapidly evolves [13]. Implementation science offers the best approach for translating the clinical recommendations made into routine practice by overcoming barriers to and leveraging enablers of improved care for [5,6].
